# Successful Use of Higher-Dose Etanercept for Multirefractory Systemic Flare of Adult-Onset Still's Disease with Liver Failure with No Response to Tocilizumab Therapy

**DOI:** 10.1155/2013/923497

**Published:** 2013-12-17

**Authors:** Taio Naniwa, Shinya Tamechika, Shiho Iwagaitsu, Shinji Maeda, Hiroyuki Togawa

**Affiliations:** ^1^Division of Rheumatology, Nagoya City University Hospital and Department of Medical Oncology and Immunology, Nagoya City University Graduate School of Medical Sciences, Kawasumi, Mizuho-ku, Nagoya, Aichi 467-8601, Japan; ^2^Department of Cardio-Renal Medicine and Hypertension, Nagoya City University Graduate School of Medical Sciences, Kawasumi, Mizuho-ku, Nagoya, Aichi 467-8601, Japan

## Abstract

A 21-year-old woman with refractory systemic flare of adult-onset Still's disease with liver failure despite high-dose corticosteroids, cyclosporine, tacrolimus, and tocilizumab, was successfully treated with additional use of etanercept. Etanercept at a dose of 50 mg weekly was partially effective but could not reduce the dose of concomitant betamethasone from 5 mg/day. Etanercept at a dose of 75 mg weekly could lead her to clinical remission and enabled successful tapering off the corticosteroids and discontinuation of etanercept. Normalization of serum C-reactive protein and interleukin 6 and persistent elevation of serum tumor necrosis factor **α** under the treatment with high-dose corticosteroids and immunosuppressants suggest that tumor necrosis factor **α** was more deeply involved than at least interleukin 6 in the pathogenesis of refractoriness of the disease in this patient, and these findings might be indicative of potential efficacy for adjunctive use of a tumor necrosis factor inhibitor rather than an interleukin 6 inhibitor.

## 1. Introduction

Adult-onset Still's disease (AOSD) is a systemic inflammatory disease characterized by spike fevers, evanescent rash, and polyarthritis [1, 2]. Although systemic flares of the disease may be usually successfully treated with high-dose corticosteroids with or without immunosuppressants, refractory systemic flares of the disease may potentially cause life-threatening conditions, such as macrophage activation syndrome and hepatic failure [1–4].

The biologic agents that selectively inhibit the action of proinflammatory cytokines such as tumor necrosis factor *α* (TNF*α*), interleukin (IL) 1, and IL6 have been successfully used for the treatment of AOSD refractory to conventional therapies that in turn reinforce the importance of excess of proinflammatory cytokines in the pathogenesis of AOSD [1, 2]. Several case reports regarding efficacy of switching biologics in AOSD suggest that effective targets for cytokine inhibitors are different among individual patients and in turn mirror the fact that cytokines with the more dominant role in maintaining the refractoriness of AOSD to conventional therapy are different [2, 5–7]. Here, we report a patient with systemic flare of AOSD with no response to treatment with high-dose corticosteroids, immunosuppressants, and anti-IL6 receptor antibody, tocilizumab, and successfully treated with higher-dose soluble TNF*α* receptor agent, etanercept, in conjunction with the results of serum levels of C-reactive protein (CRP) and proinflammatory cytokines.

## 2. Case Presentation

A previously healthy 21-year-old Japanese woman was admitted to the dermatology department in the hospital with a 2-week history of high fever, rash, and polyarthralgia. Generalized salmon pink-colored and morbilliform maculopapular eruptions with Köbner's reaction were observed. Neither lymphadenopathy nor splenomegaly was observed. Blood test showed markedly elevated white blood cell count of 24,100/mm^3^ with neutrophil at 92%, CRP of 189 mg/L, and ferritin of 12,000 ng/mL and normal alanine aminotransferase (ALT). The antinuclear antibody test was positive at 1 : 320 dilution with a homogenous pattern but anti-DNA, anti-Sm, anti-RNP, anti-Ro, and anti-Jo-1 were all negative. Rheumatoid factor was also negative. Skin biopsy revealed dermal perivascular leukocyte infiltration without liquefaction degeneration at the dermoepidermal junction. She was diagnosed as having AOSD and treated with methylprednisolone pulse therapy (1 g/day for 3 days) followed by oral prednisolone 60 mg/day; however, she relapsed fever, rashes, and developed progressive liver dysfunction and was referred to our hospital.

**Figure 1:**
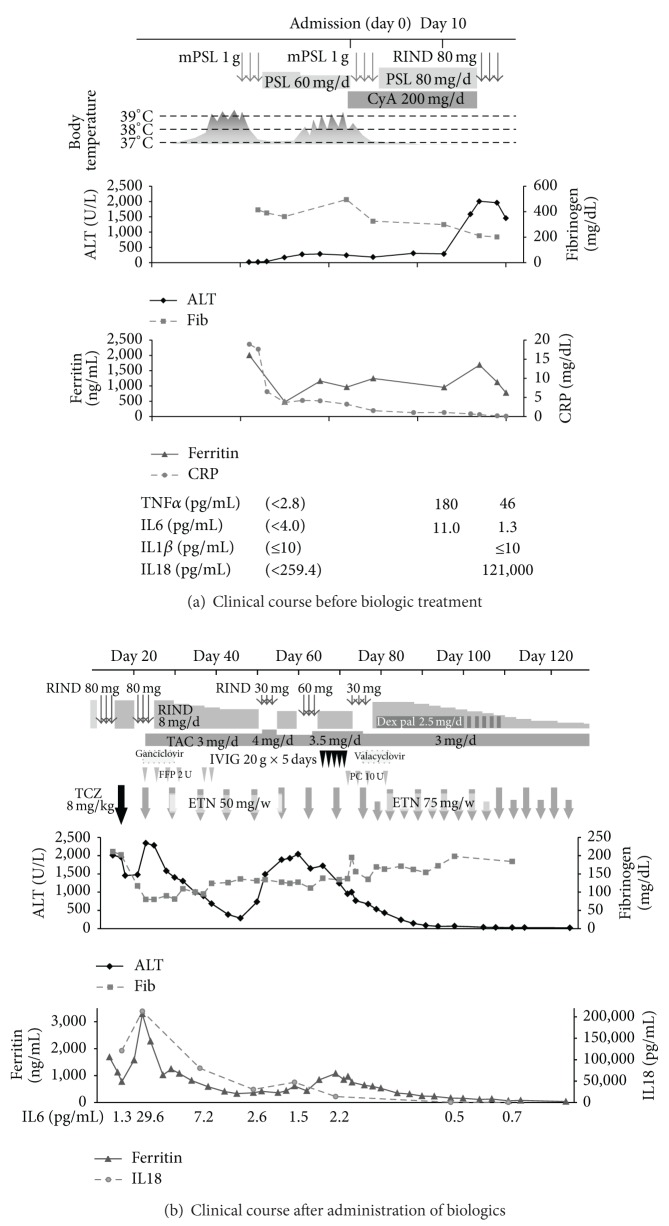
Patient's treatment, liver function, and inflammatory biomarkers during the clinical course. ALT, alanine aminotransferase; CRP, C-reactive protein; CyA, cyclosporine; Dex-Pal, dexamethasone palmitate; ETN, etanercept; HD-IVIg, high-dose intravenous immunoglobulin therapy; IFN, interferon; IL, interleukin; mPSL, methylprednisolone; PSL, prednisolone; RIND (betamethasone), sIL2-R, soluble interleukin 2 receptor; TAC, tacrolimus; TCZ, tocilizumab. Cytokines were measured by SRL Inc. with the following kits; TNF*α*, Quantikine HS Human TNF-*α* immunoassay (R&D); interferon-*γ*, IFN-*γ* Human Direct ELISA Kit (Life Technologies Japan); IL1*β*, IL-1*β* Human ELISA Kit (Life Technologies Japan); IL6 Lumipulse human IL6 (Fuji Rebio); IL18, Human IL-18 ELISA kit (MBL). Reference values are as follows: TNF*α* (normal < 2.8 pg/mL), IFN*γ* (normal < 0.1 pg/mL), IL1*β* (normal < 10 pg/mL), IL6 (normal < 4 pg/mL), IL18 (normal < 259.4 pg/mL).

On admission, elevated CRP of 3.23 mg/dL, aspartate aminotransferase (AST) of 117 U/L, ALT of 244 U/L, lactate dehydrogenase of 608 U/L, and hyperferritinemia of 958 ng/mL were observed. Serology for hepatitis A, B, and C virus, human immunodeficiency virus, human T-cell leukemia virus, and human parvovirus B19 were all negative. Polymerase chain reactions did not detect hepatitis B virus DNA, Epstein-Barr virus DNA, human parvovirus B19 DNA, and human herpes virus 6 DNA. Cytomegalovirus antigen in peripheral blood leukocytes was also negative. Under the diagnosis of refractory AOSD based on the four major criteria and the one minor criterion described in a study by Yamaguchi et al. [8] as well as exclusion of other conditions, she received pulsed methylprednisolone 1 g/day for 3 days following prednisolone 80 mg/day combined with cyclosporine 200 mg/day with trough serum concentration of 116.5 to 194.1 ng/mL which brought her afebrile state but did not inhibit progressive liver dysfunction and hyperferritinemia (Figure 1(a)). From the day 16 to 18, intravenous pulsed betamethasone 80 mg/day was added, and tocilizumab was added on the day 19. The test for cytomegalovirus antigen in peripheral blood leukocytes became slightly positive (7/50,000 cells); ganciclovir was used for 8 days from the day 22. Despite the treatment with betamethasone pulse and tocilizumab, liver function was progressively deteriorated. Fibrinogen and prothrombin time was decreased at 80 mg/dL and at 62.0%, respectively. Serum ferritin was elevated at 3,283 ng/mL on the day 24. Thus, on the same day, betamethasone 80 mg/day for 3 days was administered again, cyclosporine was substituted to tacrolimus 3 mg/day, and etanercept 50 mg/week was started. After introduction of the combination therapy including etanercept, liver dysfunction, fibrinogenopenia, and hyperferritinemia had partially improved. Betamethasone was tapered from 8 mg/day to 5 mg/day which resulted in exacerbation of liver dysfunction and hyperferritinemia. The dose of corticosteroids was increased again with 3 courses of intravenous betamethasone pulses (30 mg for 3 days, twice and 60 mg for 3 days, once) and daily intravenous dexamethasone palmitate (2.5 mg/day). High-dose intravenous immunoglobulin therapy (20 g for 5 days) was also added. As the test for CMV antigen in the peripheral blood leukocyte was slightly positive (4/50,000 cells) in the day 74, valacyclovir was administered for 8 days from the day 78. Due to inadequate efficacy of the combination therapy, we increased the dose of etanercept to 75 mg/week from the day 79. Thereafter, liver transaminases, fibrinogen, and ferritin levels became rapidly normalized and the dose of corticosteroids could be tapered rapidly (Figure 1(b)). After 7-week administration of 75 mg/week of etanercept, an attempt to reduce the dose was followed by no flare of clinical manifestations and laboratory findings; thus we discontinued etanercept which had been used for 36 weeks. Thereafter corticosteroids and tacrolimus were safely tapered off and she has been in remission on methotrexate 6 mg/week.

Analyses of serum levels of proinflammatory cytokines revealed IL6 levels had decreased and normalized after the day 19, just before tocilizumab infusion. On the other hand, serum levels of TNF*α* had decreased but remained abnormally high before tocilizumab infusion. IL18 levels were well correlated with ferritin levels and the extent of liver dysfunction during the clinical course of this patient. Serum levels of ferritin and IL18 were normalized after the onset of etanercept and maintained within normal range for 1.5 years after discontinuation of etanercept.

## 3. Discussion

Recently, off-label use of biologic agents that inhibit proinflammatory cytokines, such as TNF, IL1, and IL6, has been successful in treatment-refractory cases. Several case reports regarding efficacy of switching an anticytokine biologic to another with different mode of action in AOSD suggest that effective targets for cytokine inhibitors are different among individual patients and in turn mirrors, the fact that cytokines with more dominant role in maintaining the refractory disease to conventional therapy are different [2, 5–7]. However, it is not yet well studied about how to predict which biologic treatment will work best for a particular patient.

In the present case, serum CRP levels, which are directly regulated by endogenous IL6 production, and serum IL6 levels were normal just before administration of tocilizumab. In addition, serum IL6 level after administration of tocilizumab, which is known to increase more and reflect the actual endogenous IL6 production much better than the serum IL6 level before tocilizumab treatment [9], showed an increase at day 5 after administration of tocilizumab (29.6 pg/mL) but thereafter promptly decreased to within-normal range. In our previous case report of the AOSD patient successfully treated by tocilizumab, serum IL6 level showed a significant increase 14 days after the start of tocilizumab (166 pg/mL) compared to just before administration of tocilizumab (12.2 pg/mL), then reached the highest value after 71 days (365 pg/mL), and thereafter decreased to 43.8 pg/mL just before successful withdrawal of tocilizumab treatment [10]. Therefore, it is suggested that IL6-driven inflammation was already efficiently suppressed during the treatment with high-dose corticosteroids and cyclosporine in this patient. On the other hand, serum level of TNF*α* just before tocilizumab therapy was 180 to 46 pg/mL, which is 64- to 16-fold higher than the upper limit value of normal range (2.8 pg/mL) despite high-dose corticosteroids and cyclosporine before the biologic therapy. These findings suggest that the efficacy of etanercept in this patient reinforces the concept that TNF*α* was more deeply involved than at least IL6 in the refractory pathological condition in this patient. Serum IL1*β* level just before tocilizumab therapy was under the measurement limit in this patient; however, it should be noted that low circulating levels of a cytokine do not necessarily reflect its less importance for AOSD [11]. Therefore, normal serum levels of CRP and IL6 under the conventional treatment including high-dose corticosteroids and/or immunosuppressants might predict poor response to the IL6-targeting therapy and high serum TNF*α* level might predict efficacy of TNF-targeting therapy in refractory acute flare of AOSD patients.

Serum concentrations of IL18 correlated well with clinical activity scores and biological indicators of inflammation, such as ferritin, in patients with AOSD [4, 12, 13]. In addition, IL18 concentration was markedly increased in the patient with active hepatitis associated with AOSD and intense IL18 expression was detected within macrophages in the liver parenchyma [14]. In this patient, serum IL18 levels correlated well with serum ferritin levels and the extent of liver dysfunction but dissociated with serum CRP and IL6 levels which had remained normal after initial high-dose corticosteroids and cyclosporine therapy. A similar case with regard to dissociation between IL18 and CRP as well as IL6 was reported in which a patient was suffered from progressive liver failure requiring liver transplantation in the relapse of AOSD [4].

Another interesting point of the clinical course of this patient was that adjunctive etanercept at a dose of 50 mg/week could not lead her to remission and could not enable tapering the dose of concomitant betamethasone from 5 mg/day; however, etanercept at a dose of 75 mg/week could lead her to clinical remission and enabled successful tapering off the corticosteroids and, moreover, discontinuation of etanercept. These findings suggest that TNF*α* might be a key player forming a positive feedback circuit in maintaining the pathology of corticosteroid-refractory systemic flare of AOSD in this patient and higher-dose etanercept might be required to suppress the disease activity of refractory AOSD patients with partial response to etanercept at its approved dose for the treatment of rheumatoid arthritis.

In conclusion, this case report and relevant literature suggest that effectual targets of anticytokine therapies were different among individual patients. Serial measurement of serum levels of CRP, IL6 and TNF*α* under the conventional treatment with high-dose corticosteroid and/or immunosuppressants might mirror the more dominantly involved one between IL6 and TNF*α* in the pathogenesis of refractoriness of AOSD. In refractory systemic AOSD patients whose serum CRP and IL6 levels had normalized but hyperferritinemia and liver dysfunction had not improved and serum TNF*α* had not normalized under the conventional treatment, anti-TNF biologic might be more effective and safer treatment than at least anti-IL6 biologic as an adjunctive agent. Dose escalation of etanercept should be considered in refractory AOSD patients who had these features and partially responded to etanercept at its approved dose for the treatment of rheumatoid arthritis.
